# Global implementation and evaluation of atrial fibrillation screening in the past two decades – a narrative review

**DOI:** 10.1038/s44325-024-00014-w

**Published:** 2024-09-02

**Authors:** Kam Cheong Wong, Tu N. Nguyen, Clara K. Chow

**Affiliations:** 1 Westmead Applied Research Centre, Sydney Medical School, Faculty of Medicine and Health, The University of Sydney, Sydney, NSW Australia; 2 Westmead Clinical School, Faculty of Medicine and Health, The University of Sydney, Westmead, NSW Australia; 3 Bathurst Rural Clinical School, School of Medicine, Western Sydney University, Bathurst, NSW Australia; 4 School of Rural Health, Faculty of Medicine and Health, The University of Sydney, Orange, NSW Australia; 5 The George Institute for Global Health, Sydney, NSW Australia; 6 Department of Cardiology, Westmead Hospital, Westmead, NSW Australia; 7 Charles Perkins Centre, The University of Sydney, Sydney, NSW Australia

**Keywords:** Cardiology, Health care

## Abstract

Advances in screening technology have been made in tandem with the aging population and increasing atrial fibrillation (AF) prevalence. While several randomized controlled trials demonstrate the efficacy of AF screening, less evidence has been synthesized addressing the implementation and evaluation of AF screening programs. We systematically searched the PubMed database from 1st January 2000 to 18th January 2024. The search terms included “atrial fibrillation” and “screening” and their synonyms. Articles that described screening implementation, including screening methods, were included. Editorial, commentary, engineering, and basic science articles were excluded. 1767 abstracts were screened, of which 138 full articles were reviewed, and 87 studies were included: 90% from high-income, 8% from upper-middle-income and 2% from lower-middle-income countries/ regions. The screening initiatives included general practice (*n* = 31), remote self-screening (*n* = 30), pharmacy (*n* = 11), community centers and villages (*n* = 10), hospital (*n* = 4), and nursing home (*n* = 1). Most studies used handheld ECG devices (*n* = 72, 83%), some used wearable devices (*n* = 13, 15%), and two (2%) used implantable cardiac devices. Comparator groups were described in 17% (15/87) studies: all 6 remote self-screening trials showed superior AF detection rates compared to usual care (these studies applied intermittent screening using handheld ECG devices over 2 weeks to 12 months or wearing ECG patches for continuous monitoring over 2–4 weeks), but 9 trials using systematic and opportunistic screening in primary care settings showed mixed results. Among 72 studies without comparator groups, 18 reported new AF detection rates below 1%, 48 reported 1–10%, 5 reported above 10%, and one reported an AF incidence rate of 2.25% patient-years (95% CI 2.03–2.48). Only 22% (19/87) of studies reported on the implementation evaluation (12 by surveys and 7 by interviews), surveying participant acceptability, usability, and satisfaction, and some studies in general practice and pharmacy interviewing participants and qualitatively evaluating the enablers and barriers to implementation. These studies reported barriers of lack of resources and referral pathways and enablers of having a designated staff member to lead implementation at point-of-care settings. AF screening implementation studies were mainly conducted in high-income countries/ regions. Detection rates were highest in older and higher risk groups, and if longer continuous ECG monitoring was used. Few studies reported details of the implementation of AF screening programs concerning cost, scalability, or comparative effectiveness of remote technology-driven screening approaches versus lower-tech approaches such as pulse palpation. Despite AF screening recommendations existing for some time, we seem to lack the data to effectively scale these initiatives.

## Background

Atrial fibrillation (AF) is increasing due to population aging, increases in AF risk factors such as obesity, and partly due to increased AF detection through screening. The 2019 global burden of AF was estimated at 59.7 million people (95% confidence interval (CI) 45.7–75.3 million) and is double the estimates made in 1990^1–4^. AF is associated with a five-fold higher risk of stroke^5^, and AF-related ischemic stroke is more likely to recur and almost twice as likely to be fatal^6,7^. The total annual cost of AF treatment (including hospitalizations, inpatient cost, outpatient treatment, and prescription medications) was estimated to be US$6.65 billion in the United States of America (USA) in 2005^8^. Patients with AF utilize healthcare services more than people without AF, and in the USA, the annual average total healthcare costs for patients with AF was US$25,006 (95% CI $24,357–$26,912) more than that for patients without AF (*p* < 0.001)^9^. In Australia, the estimated cost of AF treatment was AUD881 million in 2015–2016, including hospital services, outpatient treatment, emergency departments, prescription medications and general practitioner (GP) services^10^.

**Fig. 1:**
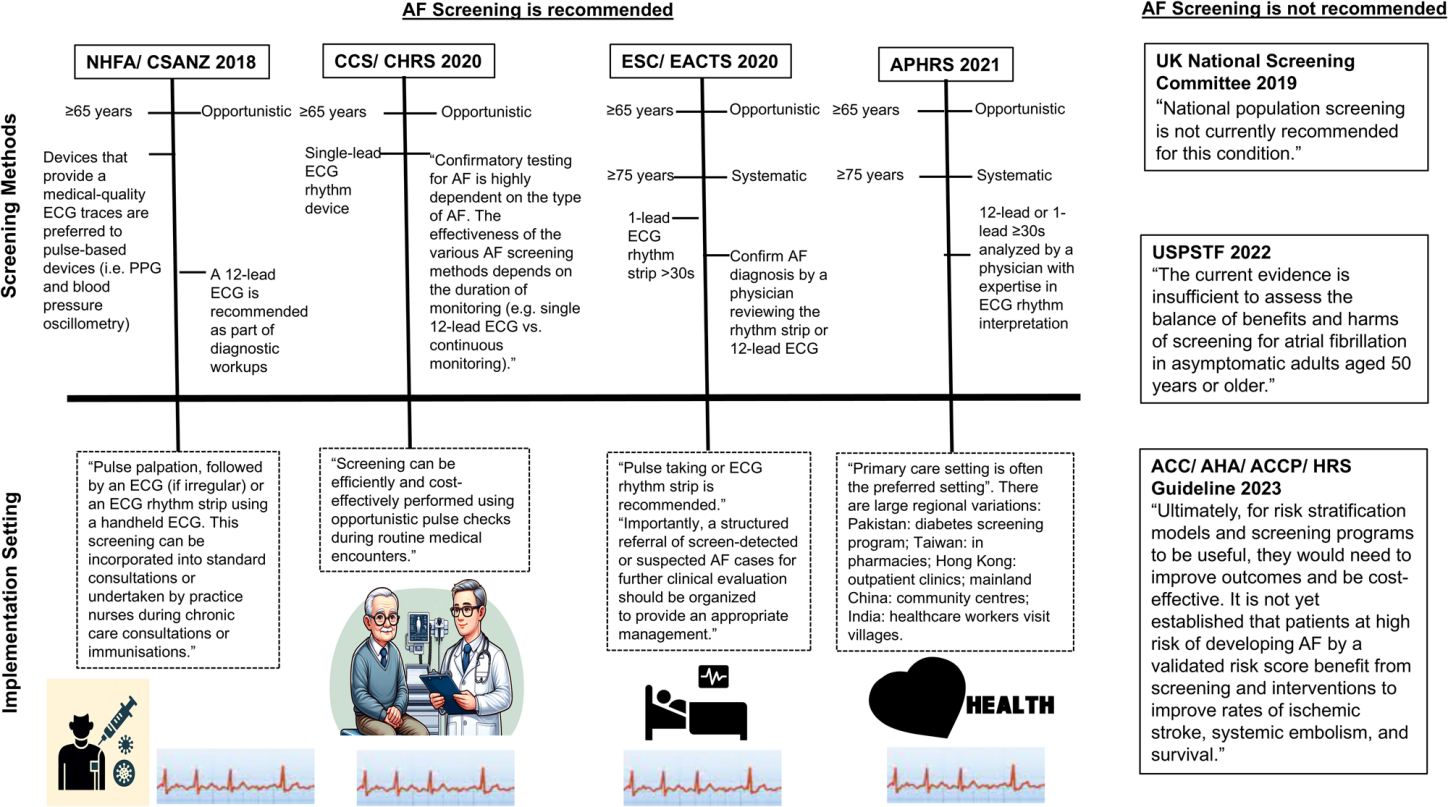
Atrial fibrillation screening guidelines in different countries and regions. ESC/ EACTS The European Society of Cardiology/European Association for Cardio-Thoracic Surgery Guidelines, CCS/ CHRS Canadian Cardiovascular Society/Canadian Heart Rhythm Society Guidelines, NHFA/CSANZ National Heart Foundation of Australia/Cardiac Society of Australia and New Zealand (CSANZ) Guidelines, APHRS Asia Pacific Heart Rhythm Society Guidelines; UK United Kingdom, USPSTF United States Preventive Services Task Force, ACC/AHA/ACCP/HRS American College of Cardiology/American Heart Association/American College of Chest Physicians/ Heart Rhythm Society Guidelines.

The increasing burden of AF on healthcare systems highlights the need for effective AF identification, prevention, and management strategies. The current recommendations for AF screening vary among Guidelines (Fig. 1). The European Society of Cardiology (ESC)/European Association for Cardio-Thoracic Surgery (EACTS)^11^, Canadian Cardiovascular Society (CCS)/Canadian Heart Rhythm Society (CHRS)^12^, National Heart Foundation of Australia (NHFA)/Cardiac Society of Australia and New Zealand (CSANZ)^13^ and Asia Pacific Heart Rhythm Society (APHRS)^14^ recommend opportunistic screening among people aged ≥65 years by pulse palpation and using electrocardiographic (ECG) devices, including the use of single-lead rhythm traces. In addition, the ESC guidelines and APHRS recommend systematic screening of people aged ≥75 years^11,14^. However, in 2019, the United Kingdom (UK) National Screening Committee issued a statement of not recommending a national population screening programme for AF because it is not clear if different types of AF have the same risk for strokes, the effectiveness of treatment for screening-detected AF is unknown, and it is not known if screening is more beneficial than the current approach to detection and management^15^. Recently, the American College of Cardiology (ACC)/American Heart Association (AHA) Joint Committee stated that it is not yet established that patients at high risk of developing AF would benefit from screening and interventions to improve rates of ischemic stroke, systemic embolism, and survival^16^. Similarly, the United States Preventive Services Task Force (USPSTF) stated that there is not enough evidence to recommend screening for adults aged 50 years or older given the potential harms of therapy – primarily bleeding from anticoagulants^17^, but the USPSTF notes that AF patients likely would benefit from contextualized management of modifiable risk factors^17–20^.

Screening for AF enables early diagnosis and allows patients to gain the potential benefits of prevention management. In many countries, cardiovascular and other chronic health condition screenings have been the domain of primary care providers (i.e., general practitioners), as has the ongoing management of high-risk AF patients. This review aims to examine and synthesize findings from AF screening studies across various settings to gain insight into screening approaches, technology use, AF detection rates, implementation, and evaluation of the screening process.

## Methods

**Fig. 2:**
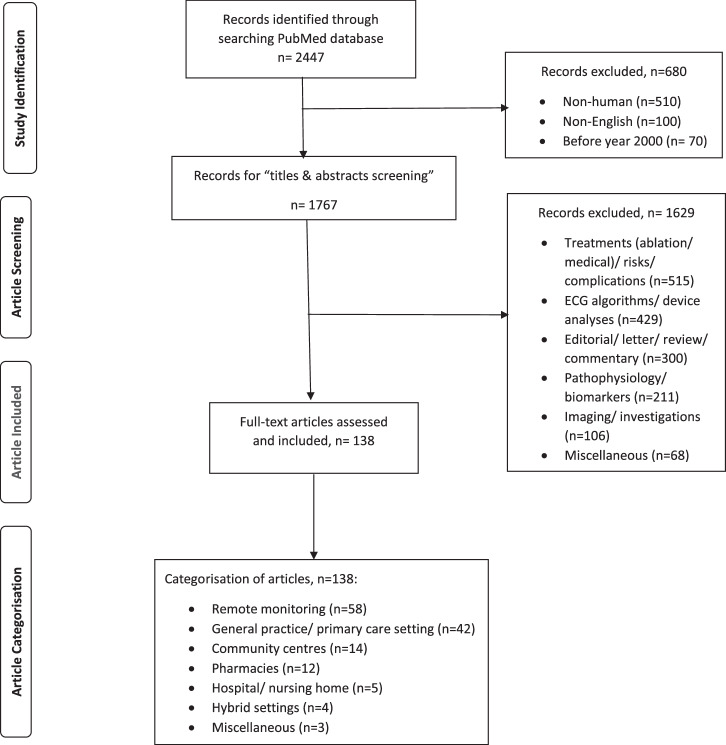
Flow diagram of the literature search process and the results of searches. ECG electrocardiogram, *n* number of articles.

The PubMed database was searched from 1st January 2000 to 18th January 2024. The search terms included “atrial fibrillation”, “auricular fibrillation”, “atrial flutter”, “screen”, “screening”, “search”, “searching”, “detect”, “detecting”, “detection”, “diagnose”, “diagnosing”, “diagnosis”, “diagnoses”, “identify”, “identifying”, and “identification”. All searches were restricted to human subjects and articles published in English (see the literature search strategies in Supplementary Table 1). Articles that described the implementation of screening programs, including the target population, screening methods (e.g. observational studies, interventional studies with or without a comparator/control) and primary outcomes, were included. Editorial, commentary, engineering articles (e.g. analysis of ECG signal processing, device accuracy studies), basic science articles (e.g. AF pathophysiology, biomarkers), and studies on post-stroke rhythm monitoring/treatments/complications were excluded (Fig. 2). The following information was extracted from the included articles and documented in Tables 1 and 2: country where the study was conducted, first author’s name, year of publication, year commenced AF screening, sample size, age eligibility (target population), risk factors included as enrollment criteria, screening methods and devices, primary outcomes, and the screening initiatives (e.g. primary care/pharmacy/remote screening or hospital and other community initiatives). The countries or regions where the studies were conducted were graphically identified on a world map^21^.

**Table 1:** Atrial fibrillation screening studies with intervention and comparator groups

Country/region, first author, ref.	Sample size	Age eligibility (years)	Screening subjects, devices, and methods	Primary outcomes
**General practice/primary care screening initiatives**				
UK, Morgan^46^ - RCT	3001	≥65	Intervention: systematic invitation of eligible patients; nurses performed pulse palpation followed by a confirmatory 12-lead ECG. Comparator: opportunistic case-finding using visual cues (in paper or computer records) to prompt GPs to perform pulse palpation.	AF: 67 (4.5%) in the intervention group and 19 (1.3%) in the comparator (Difference = 3.2%, 95% CI = 2.0–4.4%).
UK, Hobbs^41^, Fitzmaurice^124^ - RCT	14,802	≥65	Intervention (a) systematic invitation for ECG or (b) opportunistic screening (pulse palpation by GPs followed by 12-lead ECG if the pulse was irregular. The screening took place over 12 months. Comparator: Usual care, no active screening.	AF: 1.63% a year (intervention practices) and 1.04% (comparator) (Difference 0.59%, 95% CI 0.20–0.98%).
Spain, Benito^29^ - RCT	928	≥65^b^	Potential participants (with one or more of these conditions: arterial hypertension, ischemic heart disease, valvular heart disease, diabetes, and/or congestive heart failure) were systematically identified from the residents of a primary healthcare center in an urban area. Intervention (*n* = 463): Visit practice once every 6 months over 2 years, and an ECG and physical examination were performed. Control (*n* = 465): usual care.	At 6 months, 8 AF in the intervention and 1 in the control (1.7% vs. 0.2%, *P* = 0.018). After 2 years, 11 AF in the intervention and 6 in the control (2.5% vs. 1.3%, *P* = 0.132).
Spain, González^39^ - RCT	6990	≥65	Opportunistic screening group (*n* = 5465): GPs performed pulse palpation followed by a 12-lead ECG if the pulse was irregular. Case finding group (*n* = 1525): actively searched for patients with symptoms of AF.	New AF: 6.7% in the Case Finding group and 1.1% in the Opportunistic Screening group.
Netherlands, Kaasenbrood^42^ - RCT	919	≥65	GPs opportunistically screened eligible patients using a 1-lead ECG device (MyDiagnostick). Control: usual care.	New AF: 1.43% (intervention) and 1.37% (usual care) (*P* = 0.73).
Netherlands, Uittenbogaart^60^ - RCT	18,744	≥65	Intervention (*n* = 9218): GPs opportunistically screened eligible patients using pulse palpation, Sphygmomanometer with AF-detecting algorithm, and 1-lead ECG (MyDiagnostick) followed by 12-lead or Holter per the protocol. Control (*n* = 9526): usual care.	New AF: 144 (1.62%) in the intervention and 139 (1.53%) in the usual care group (OR 1.06, 95% CI 0.84–1.35).
China, Chen^32^, Zhang^61^ Not-RCT	4531	≥65	Participants were recruited via social media to attend screening using 1-lead ECG (Kardia) overread by cardiologists. Participants were randomized into (a) annual screening or (b) quarterly screening groups.	Overall AF prevalence: 183 (4.0%). New AF: 6.7 per 1000 person-years (quarterly screening) and 4.1 per 1000 person-years (annual screening) (Hazard Ratio 1.71; 95% CI 1.06–2.76; *p* = 0.029).
USA, Lubitz^44^ - RCT	30,715	≥65	Intervention (*n* = 15,393): clinic medical assistants opportunistically screened patients using 1-lead ECG (Kardia) during vital sign assessments. Control (*n* = 15,322): usual care.	AF: 1.72% in the intervention and 1.59% in the control (*P* = 0.38).
Spain, Cabrera^31^ - RCT	2231	≥65^b^	Eligible individuals with (one or more risk factors: hypertension, diabetes, ischemic or valve heart disease, heart failure and obstructive sleep apnoea) were systematically identified and invited. Intervention (*n* = 728): participants had an ECG at 0, 6, and 12 months or the GP auscultated an arrhythmia. Control (*n* = 1503): usual care.	New AF: 20 (2.7%) in the intervention group and 18 (1.2%) in the control (Hazard Ratio 2.39, 95% CI 1.26–4.53, *P* = 0.007).
**Remote self-screening**				
Sweden, Svennberg^112^: “STROKESTOP 1 - RCT	7173	75–76	In the intervention, eligible individuals were systematically invited to self-screen for 14 days using 1-lead ECGs (Zenicor) twice daily and when they had palpitations. Individuals in the control received the usual care.	New AF: 218 (3.0%; 95% CI, 2.7–3.5%), 4-fold higher compared to control usual care group.
UK, Halcox^99^ - RCT	1001	≥65^b^	Eligible patients, with a CHADS-VASc score ≥2, were systematically searched from GPs’ records and invited to self-screen using 1-lead ECGs (Kardia) twice weekly over 12 months and an additional recording if symptomatic. The control group received the usual care.	New AF: 19 (3.8%, intervention), 5 (1.0%), control), (Hazard ratio, 3.9; 95% CI = 1.4–10.4; *P* = 0.007).
USA, Steinhubl^110^ - RCT	2659	Varied age^a,b^	Members of a large national insurance health plan were invited to self-screen by wearing a continuous ECG monitoring patch (Zio) for up to 4 weeks. Intervention: commence immediately after enrolling; Waitlist control: delayed for 4 months after enrollment.	At 4 months, new AF: 3.9% (53/1366) of the immediate intervention group vs 0.9% (12/1293) in the delayed group (absolute difference, 3.0%, 95% CI, 1.8–4.1%).
Canada, Germany, Gladstone^92^ (The first author was from Canada) - RCT	856	≥75^b^	Eligible individuals with hypertension were opportunistically recruited from general practices to self-screen by wearing Zio patch for 14 days at baseline and another patch at 3 months. The control group received the usual care.	New AF: 23/434 (5.3%, intervention) vs 2/422 (0.5%, control) (relative risk, 11.2; 95% CI, 2.7–47.1; *P* = 0.001).
Denmark, Svendsen^111^: “LOOP Study” - RCT	6004	70–90^b^	Hospital patients aged 70–90 years with at least one stroke risk factor (ie, hypertension, diabetes, previous stroke, or heart failure) were randomly assigned to implantable loop recorder (ILR) monitoring (*n* = 1501) or usual care (control, *n* = 4503), for various follow up times (median= 64.5 months).	New AF: 477 (31.8%, ILR group) vs 550 (12.2%, control group) (hazard ratio 3.17, 95% CI 2.81–3.59; *P* < 0.0001).
Germany, Rizas^107^ - RCT	5551	≥50^b^	Insurance policyholders aged 50–90 years or with a CHA2DS2-VASc score of ≥1 (men) or ≥2 (women), were systematically identified and invited to participate in self-screening using a PPG smartphone app (Preventicus Heartbeats) twice daily for the first 14 days and twice weekly thereafter (*n* = 2860). Abnormal findings were evaluated by 14-day external ECG loop recorders. The control group received the usual care (*n* = 2691). After 6 months, participants were crossed over between the groups.	Screening-detected AF versus usual care-detected AF: odds ratios 2.12 (95% CI 1.19–3.76; *P* = 0.010, before cross over) and 2.75 (95% CI 1.42–5.34; *P* = 0.003, after cross over).

*RCT* randomized control trial.

^a^Varied age: individuals age ≥75 years old, or male age >55 years, or females age >65 years, with one of the following morbidities: prior stroke or transient ischemic attack, heart failure, diagnosis of both diabetes and hypertension, mitral valve disease, left ventricular hypertrophy, or Chronic Obstructive Pulmonary Disease requiring home oxygen, sleep apnea, history of pulmonary embolism, history of myocardial infarction, or diagnosis of obesity.

^b^Risk factor(s) was/were added in addition to eligible age as enrollment criteria.

**Table 2:** Atrial fibrillation screening studies without a comparator group

Country/region, first author, ref.	Sample size	Age eligibility (years)	Screening subjects, devices, and methods	Primary outcomes
**General practice/ primary care screening initiatives**				
UK, Somerville^57^	86	≥65	Participants with and without AF (ratio 1:1) were systematically searched from the medical records in a single general practice and invited to screen. Nurses performed pulse palpation followed by a 12-lead ECG (if the pulse was irregular).	Overall AF: 26 (30%) (included patients with a prior diagnosis of AF).
Belgium, Claes^33^	10,758	≥40	Participants were invited via social media (radio, newspaper, magazines) to participate in a nationwide screening in 69 medical centers. Nurses performed screening using 1-lead ECGs (HeartScan, Omron).	AF prevalence of 2.2% (95% CI 1.3% and 3.0%); The age of AF patients: 67 ± 12 y.
UK, Rhys^52^	573	≥65	Participants attending annual flu vaccination clinics were opportunistically offered screening by pulse palpation followed by a 12-lead ECG if the pulse was irregular.	New AF: 2 (0.4%), and 21 had a prior diagnosis of AF.
Spain, Sanmartin^54^	1532	≥65	Participants were identified by systematically searching the medical records and invited via letters. Nurses performed pulse palpation and a 12-lead ECG if an irregular pulse was detected.	1532 individuals were evaluated (mean age: 72.5 (SD 6.5) years), 187 patients had ECGs and 17 new AF (1.1%).
Australia, Orchard^47^	88	≥65	Patients attending the general practices were opportunistically invited. Receptionists or nurses screened patients using a 1-lead ECG (Kardia), and GPs discussed the device algorithm’s automatic results with the patients. Semi-structured interviews were conducted to evaluate feasibility.	17 patients (19%) were in AF (all previously diagnosed).
Ireland, Bury^30^	566	≥70	26 randomly selected general practices systematically identified patients from their databases (and excluded known AF) to attend the screening. A 2-min 3-lead ECG was acquired and collected centrally for cardiologist review.	New AF: 12 (2.1%) (mean age 79, range 73–88 years).
Italy, Salvatori^53^	274	≥65^c^	Potential participants with hypertension were systematically identified from the GP database. Participants had baseline 12-lead ECG; if this did not show AF, 48-h Holter monitoring was performed.	New AF: 27 (10%; 95% CI 6.4–13.5%).
Australia, Orchard^49^	973	≥65	Patients attending annual flu vaccination were opportunistically offered the screening using 1-lead ECG (Kardia). Semistructured interviews were conducted with nurses, practice managers and GPs.	New AF: 8 patients (0.8%).
Netherlands, Kaasenbrood^43^	3269	≥60	Individuals attending flu vaccination were opportunistically invited to record a 1-lead ECG (MyDiagnostick). A cardiologist analyzed ECGs with suspected AF.	New AF: 37 (1.1%) with a mean age of 75.9 (SD 8.6) years.
Ireland, Smyth^56^	7262	≥65	GPs were asked to systematically screen consecutive eligible patients by pulse palpation followed by a 12-lead ECG if the pulse was irregular.	916 (12.6%) had irregular pulses, of whom 735 (10.1%) had known AF and 55 (0.8%) had newly detected AF.
Denmark, Hald^40^	970	≥65	Nurses performed pulse palpation followed by a 12-led ECG if the pulse was irregular. Two cardiologists validated all ECGs.	87 (9%) patients had an irregular pulse, and 10 patients (1.0%) had new AF.
Ecuador, Del Bruto^34^	298	≥60	Systematically invited eligible individuals living in a rural town to have a 12-lead ECG at enrollment followed by 24-h Holter monitor.	New AF: 7 (2.3%) (none of them had AF in the 12-L ECG at enrollment).
Australia, Orchard^48,50,51^ – AF SMART Study	4908	≥65	Eligible patients were identified using electronic screening prompts and screened using 1-lead ECG (Kardia) by GPs in urban (*n* = 1805) and rural (*n* = 3103) areas.	New AF: in urban, 19 (1.1%) (mean age, 75.7 years); in rural, 36 (1.2%) (mean age, 75.1 years).
Canada, Godin^38^	7585	≥65	GPs were asked to opportunistically screen eligible patients using 1-lead ECG (Kardia). GPs were surveyed on their acceptability of the device.	New AF: 471 (6.2%).
Japan, Suzuki^59^	9921	≥65	GPs opportunistically screened eligible patients by pulse palpation followed by an ECG when an irregular pulse was detected.	New AF: 86 (0.9%).
Canada, Andrade^27^	16,817	≥65	GPs opportunistically screened their patients using 1-lead ECG (Kardia) as part of their routine consultations.	New AF: 1171 (7.0%).
Spain, Ballesta-Ors^28^	29,597	≥60	GPs opportunistically performed pulse palpation followed by a 12-lead ECG if the pulse was irregular.	New AF: 765 (2.6%).
Brazil, Diamantino^36^	1518	Not specified	Primary care patients waiting for echocardiogram were opportunistically invited to the screen using 1-lead ECG (MyDiagnostick). AF was confirmed using a 12-lead ECG.	AF: 97 (6.4%).
Italy, Denas^35^	14,987	≥65	A population-based prospective study systematically searched and invited eligible individuals to the screening using a sphygmomanometer embedded with an AF-detecting algorithm (WatchBP, Microlife).	AF incidence rate: 2.25% patient-years (95% CI 2.03–2.48%).
USA, Stavrakis^58^	1019	≥50	Eligible individuals who attended the Indigenous health center were opportunistically screened using 1-lead ECG (Kardia).	New AF: 15 (1.5%) (mean age 65.9 ± 10.3 years).
Brazil, Santos^55^	13,260	Not specified	Civil servants attending yearly health assessments were opportunistically screened using 12-lead ECGs.	New AF: 333 (2.5%).
Australia, Giskes^37^, McKenzie^45^	1127	≥65	Eligible patients visiting GPs were opportunistically invited to record 1-lead ECG (Kardia) at waiting rooms. ECGs with automated results were transmitted to patients’ medical records for GPs to discuss with patients.	New AF: 49 (4.3%)
**Pharmacy screening initiatives**				
Australia, Lowres^67,68^	1000	≥65	Eligible individuals visiting the pharmacies were opportunistically screened using 1-lead ECG (Kardia) over-read by cardiologists.	15 AF cases; AF detection rate 1.5% (95% CI, 0.8–2.5%); mean age 79 ± 6 years.
New Zealand, Walker^72^	121	≥55	Individuals, including Maori and Pacific people who visited the pharmacy, were opportunistically screened using 1-lead ECG (Kardia) over-read by cardiologists.	New AF: 2 (1.7%)
UK, Twigg^70^	594	Varied age^a,c^	Eligible people were opportunistically screened using a sphygmomanometer with an AF-detecting algorithm and a 1-lead ECG. Traces with possible AF were remotely reviewed and confirmed by a cardiologist.	New AF: 5 (0.8%)
Italy, Bacchini^63^	3071	≥50	Eligible were opportunistically screened using a sphygmomanometer embedded with an AF-detecting algorithm (Microlife AFIB).	Overall AF diagnosis: 98 (3.1%) (of which 54 (1.8%) were new AF).
10 countries, Da Costa^66^ (The first author was from Portugal)	2762	≥40	Pharmacists performed pulse palpation and confirmed using 1-lead ECG (Kardia). Pharmacists referred patients with possible AF to physicians.	New AF: 5 (0.2%).
USA, Anderson^62^	697	Not specified	Pharmacy students opportunistically screened people at health fairs using 1-lead ECG (Kardia).	Possible AF: 16 (2.3%) (Did not confirm AF)
Poland, Zaprutko^73^	525	≥65	Eligible individuals were opportunistically screened using 1-lead ECG (Kardia) remotely reviewed by cardiologists.	New AF: 7 (1.3%).
Portugal, Cunha^65^	205	≥40	Pharmacist opportunistically screened eligible individuals using 1-lead ECG (Kardia). AF was confirmed using 12-lead ECG interpreted by a cardiologist.	New AF: 14 (6.8%).
UK, Savickas^69^	604	≥65	Pharmacists opportunistically screened eligible patients attending flu vaccination in GP practices using 1-lead ECGs (Kardia). AF was confirmed using 12-lead ECGs interpreted by cardiologists.	New AF: 4 (0.7%).
Spain, Valdivieso^71^	452	≥50	Pharmacist opportunistically screened eligible individuals using sphygmomanometer embedded with AF-detecting algorithm. Participants with suspected AF were referred to their GPs for further evaluation.	New AF: 41 (9.1%).
USA, Bleske^64^	63	Not specified	Pharmacy students opportunistically screen individuals visiting pharmacies using 1-lead ECG (Kardia).	Possible AF: 3 (4.7%) (Did not confirm AF)
**Hospital/ nursing home screening initiatives**				
Belgium, Proietti^119^	65,747	≥18	Participants were opportunistically recruited from ‘Belgian Heart Rhythm Week’ campaign. Nurses performed screening using 1-lead ECG (Omron, HeartScan).	New AF: 603 (1.1%, 95% CI 0.9–1.3%).
Kenya, Evans^118^	50	Not specified	Patients in a rural hospital were opportunistically screened using 1-lead ECG (Kardia). A cardiologist interpreted the ECGs.	New AF: 4 (8%, 95% CI: 3–19%).
Belgium, Tavernier^121^	214	Not specified	Geriatric patients in a hospital were opportunistically screened using 1-lead ECGs (MyDiagnostick) that were interpreted by electrophysiologists.	New AF: 28 (13%).
USA, Rosenfeld^120^	772	Not specified	Patients in the Internal Medicine ward were opportunistically screened using 1-lead ECGs (Kardia).	Possible AF: 17 (2.2%). (Did not confirm AF)
USA, Wiesel^122^	101	≥65	Eligible nursing home residents were systematically identified and invited to screen using a sphygmomanometer with an AF-detecting algorithm (Microlife WatchBP). AF was confirmed by using a 12-lead ECG interpreted by a cardiologist.	New AF: 7 (6.9%, 95% CI 3.0–14.2%).
**Community screening initiatives**				
Italy, Omboni^81^	220	≥18	Screening team visited villages. Eligible villagers were opportunistically screened using a sphygmomanometer with an AF-detecting algorithm (Microlife WatchBP) followed by a 1-lead ECG (Cardio-A Palm ECG) interpreted by the doctor.	New AF: 4 (1.8%)
Hong Kong, Chan^74^	13,122	≥18	Eligible individuals in the community were screened by trained volunteers using 1-lead ECGs (Kardia) that a cardiologist interpreted.	New AF: 101 (0.8%).
Hong Kong, Chan^75^	10,735	≥50	The “AFinder” program was implemented by a non-governmental organization screening eligible individuals using 1-lead ECGs (Kardia) that cardiologists interpreted.	New AF: 74 (0.69%; 95% CI 0.54–0.84%).
India, Soni^84^	2074	≥40	Eligible villages were systematically identified by their age and sex. Health workers visited villages and screened eligible individuals using 1-lead ECGs (Kardia) that cardiologists interpreted.	New AF: 33 (1.6%).
Japan, Nagata^80^	88,218	Not specified	Hokuriku Health Service Association in Toyama performed annual health assessments, including a 12-lead ECG, on workers and their families. Eligible individuals were systematically identified from their records.	Total AF: 415 (0.47%), of which 69 (0.08%) were new AF).
Australia, Gwynn^77^	619	≥45	Eligible Aboriginal people were opportunistically screened using 1-lead ECGs (Kardia) that a cardiologist interpreted.	Total AF: 29 (4.7%; 95% CI 3.0–6.4%), of which 4 (0.6%) were new AF.
Australia, Jatau^79^	1704	≥65	Screening stations were set up in community venues to opportunistically screen eligible individuals using a sphygmomanometer with an AF-detecting algorithm (Microlife WatchBP).	Total AF: 22, of which 16 (0.9%) were new AF.
Japan, Senoo^83^	1607	≥65	Eligible people in the community were systematically identified and invited to a community screening using a sphygmomanometer attached with a 1-lead ECG (Complete, Omron) that cardiologists interpreted.	New AF: 15 (0.93%).
Denmark, Poulsen^82^	477	≥75	Caregivers performed home visits to eligible residents and opportunistically screened them using 1-lead ECG (Zenicor) that were remotely and centrally reviewed by a cardiologist.	New AF: 7 (1.5%, 95% CI 0.6–3.0%).
Taiwan, Chao^76^	23,572	Varied age^b^	Participants in health check programs in communities were opportunistically screened using 1-lead ECGs (Rooti) interpreted by an electrophysiologist.	New AF: 278 (1.2%).
**Remote self-screening**				
Italy, Ricci^106^	166	Not specified	Participants (mean age 73 ± 10 years) had implanted monitoring devices (pacemakers or implantable cardioverter defibrillators). A nurse reviewed the reports of the devices and alerted the physician of new AF.	New AF: 22 (13.3%).
Sweden, Engdahl^89^	403	75–76^c^	All individuals who had ≥2 risk factors according to CHADS2 were systematically invited to self-screen for 2 weeks using a 1-lead ECG (Zenicor) twice daily and when palpitations occurred.	New AF: 30 (7.4%; 95% CI, 5.2–10.4%).
USA, Wiesel^116^	139	≥65	Participants were opportunistically identified and screened at home using a sphygmomanometer with an AF-detecting algorithm (Microlife BP) and a 1-lead ECG (Heartrak 2). BP findings and ECGs were transmitted to the monitoring center.	Total AF: 16, of which 2 were new AF (1.4%).
Sweden, Hendrikx^100^	928	Not specified^c^	Out-of-hospital patients without known AF who had one or more risk factors (congestive heart failure, hypertension, age ≥75 years, diabetes, and previous stroke) were opportunistically invited to self-screen using 1-lead ECGs (Zenicor) twice daily and when having palpitations over 4 weeks.	New AF: 35 (3.8%, 95% CI 2.7–5.2%).
USA, Turakhia^113^	75	≥55^c^	Eligible outpatients who had ≥2 risk factors (coronary disease, heart failure, hypertension, diabetes, and sleep apnoea) were opportunistically invited to self-screen by wearing a Zio patch for 2 weeks. An electrophysiologist confirmed AF diagnoses.	New AF: 4 (5.3%).
Germany, Busch^87^	1615	20–79	Volunteers from a general population self-screen for 4 weeks using a handheld tele-ECG card (Sensor Mobile 100) daily and when symptomatic. Physicians remotely and centrally interpreted ECGs.	New AF: 43 (2.7%) through the tele-ECG monitoring over a median follow-up of 6.3 years.
Norway, Berge^85^	1510	65^c^	Eligible individuals (CHA2DS2-VASc score ≥2 for men and ≥3 for women) were systematically identified and invited to self-screen for 2 weeks using 1-lead ECGs (Zenicor) twice daily and when symptomatic.	New AF: 13 (0.9%, 95% CI 0.5–1.5%).
Sweden, Mandalenakis^103^	798	≥50	Eligible participants had physical examinations, including a 12-lead ECG in 1993, 2003 and 2014. In 2014, participants without AF were given a 1-lead ECG (Zenicor) to self-screen twice daily over 2 weeks. The ECGs were electronically sent to the study center and overread by the researchers.	During a 21-year follow-up, 77 (9.6%) AF. AF incidence increased from 2.2 per 1000 years at the age of 50–54 years to 9.3 per 1000 years at the age 65–70 years.
Sweden, Ghazal^91^	324	70–74	Eligible individuals were systematically identified from a primary care center and invited to self-screen for 2 weeks using 1-lead ECGs (Zenicor) twice a day and if palpitations were present.	New AF: 16 (5.5%).
Japan, Fukuma^90^	100	Not specified minimal eligible age^c^	Eligible employees with a CHADS2 score ≥1 in an organization were opportunistically invited to self-screen by wearing a T-shirt embedded with a 1-lead ECG for ≥4 days/week over 2 months. Cardiologists interpreted all ECGs.	New AF: 10 (10%).
Belgium, Verbrugge^114^	12,328	Not specified	An advertisement in the local newspaper gave participants a free QR-code to download an App to their smartphones and self-screen for 7 days using photoplethysmography (PPG) twice daily and when symptomatic.	Possible AF: 136 (1.1%).
China, Guo^94^: “mAFA I”	187,912	≥18	Participants wore watches/bands with PPG technology (Huawei) for 14 days. PPG-detected possible AF were confirmed by ECGs or 24-h Holter monitors.	mAFA I Suspected AF: 424 (0.23%), 262 were effectively followed up, of which 227 were confirmed AF.
China, Guo^95,96^: “mAFA II”	2,852,217	≥18	The screened-detected AF patients were offered mobile-health-technology-supported AF integrated care aimed to avoid stroke with anticoagulants, better symptom management, cardiovascular and other comorbidities risk management, with educational programs.	mAFA II Suspected AF: 12,244 (0.43%), 5,227 were effectively followed up, of which 4903 were confirmed AF.
USA, Perez^105^: “The Apple Heart Study”	419,297	≥22	Participants wore Apple Watches with PPG sensors. When an irregular pulse was detected, participants had a telemedicine visit and were sent an ECG patch to be worn for up to 7 days.	Irregular pulse: 2,161 (0.52%), of which 450 returned patches, and 153 were confirmed AF.
Canada, McIntyre^104^	100	≥80^c^	Eligible individuals who had hypertension and ≥1 of the following: diabetes mellitus, BMI ≥ 30, sleep apnea, history of smoking, coronary disease, heart failure or left ventricular hypertrophy were recruited from primary care clinics to self-screen by wearing a 1‑lead ECG (Vitaphone 3100) for 30 days.	New AF: 14 (14%).
Greece, Savvari^109^	2408	≥50^c^	Eligible hypertensive patients visiting cardiologists and hospital hypertension clinics had 12-lead ECG. They were opportunistically given a sphygmomanometer with an AF-detecting algorithm (Microlife BP) to self-screen twice daily for 7 days, then returned to the physicians for evaluation.	AF: 293/2408 (12.5%)
Russia, Gognieva^93^	3249	≥20^c^	Eligible individuals visiting primary care centers and had one or more risk factors: hypertension, history of ischemic stroke or transient ischemic attacks, diabetes, obesity, heart failure or decreased physical activity due to dyspnea, coronary artery disease (CAD) or chest pain without established CAD diagnosis, peripheral arterial disease, abnormal heart rhythm (episodes of palpitations, pauses in heartbeat). Participants self-screen using 1-lead ECGs (CardioQVARK) over 18 months. Cardiologists confirmed AF diagnoses.	New AF: 36 (1.1%).
Poland, Kalarus^101^	3014	≥65	Eligible individuals were systematically identified and invited to self-screen by wearing a two-lead ECG vest for up to 30 days. AF episodes that lasted longer than 30 s were detected and confirmed by cardiologists.	New AF: 4.1% (95% CI, 3.5–4.8%).
Germany, Birkemeyer^86^	215	≥65	Eligible participants were opportunistically invited to self-screen for 14 days using a smartphone application (PPG algorithm, Preventicus Heartbeats) to record two readings over 1 min daily.	New AF: 3 (1.4%).
USA, Lubitz^102^: “Fitbit Heart Study”	455,699	≥22	Participants wore Fitbit devices (with PPG sensors). When an irregular pulse was detected, participants had a telehealth visit and were sent a 1-lead ECG patch (ePatch, BioTelemetry, Inc.) to be worn for up to 7 days.	Irregular pulse: 4728 (1%), of which 1057 returned analyzable patches, and 340 were confirmed AF.
Japan, Watanabe^115^	1148	≥65^c^	Eligible hospital patients with CHA 2DS2-VASc score ≥2 or CHADS2 score >1 were systematically identified and invited to use an oscillometric BP monitor (Omron) twice daily for 2 weeks. Abnormal findings were confirmed using 12-lead ECG. If the BP monitor finding was false positive, participants self-screened using a 1-lead ECG (OMRON) twice daily for an additional 2 weeks.	New AF: 9 (0.8%), of which 7 by 12-lead ECG at baseline, 1 by BP meter and 1 by 1-lead ECG.
Spain, Ximenez-Carillo^117^	600	≥70^c^	Eligible participants with at least 1 major criterion (BMI ≥ 30, hyperthyroidism, or heart failure) or 2 minor criteria (hypertension, diabetes mellitus, female, ischemic stroke, previous transient ischemic attack or systemic embolism, chronic obstructive pulmonary disease, dyslipidemia, ischemic heart disease, peripheral artery disease) were systematically identified from a primary care database and invited to self-screen by wearing a continuous monitoring band (Nuubo) for 2 weeks. AF diagnoses were confirmed by cardiologists.	New AF: 17 (2.83%).
Norway, Sandberg^108^	1849	≥65^c^	Volunteers age ≥65 years, and with minimum one other risk factor for stroke according to the CHA2DS2-VASc risk score, were invited to self-screen via social media to wear an ECG patch (ECG247) for 14 days.	New AF: 41/1849 (2.2%).
Singapore, Cai^88^	355	21–85^c^	Outpatients aged 21–85 or with CHA2DS2-VaSc ≥1 were systematically identified and invited to wear an ECG patch (Spyder ECG) for 3–14 days.	New AF: 6 (1.7%).

^a^Varied age: individuals aged ≥65 years or aged 50–64 years with one or more of the following conditions: hypertension, heart failure, raised cholesterol, pulmonary embolism, asthma/COPD, diabetes or aged 50–64 years with 2 or more lifestyle risk factors: high consumption of alcohol, smoker and Body Mass Index of 25 kg/m^2^.

^b^Varied age: residents in Yilan county aged 30–39 years, residents in Keelung county ≥30 years, and residents in Chiayi county aged ≥65 years.

^c^Risk factor(s) was/were added in addition to eligible age as enrollment criteria.

Opportunistic screening was defined as individuals visiting a setting or healthcare facility for other purposes but were opportunistically invited to participate in AF screening, while systematic screening was defined as systematically identifying individuals from a source (e.g. registry, clinical database, population database) and inviting them to screening.

Studies that reported any of the following activities were considered having evaluated the implementation: surveys, interviews or some forms of evaluation and reported acceptability, usability, use of health services triggered by notifications of potential screening abnormalities, feasibility (e.g. barriers and enablers), or process evaluation framework (e.g. the Medical Council Guidance on Process Evaluation^22^, Realist approach^23^, Critical Realism approach^24^, or the “Reach, Effectiveness, Adoption, Implementation, and Maintenance of interventions, RE-AIM” approach^25^). The results of this review were narratively summarized.

## Results

**Fig. 3:**
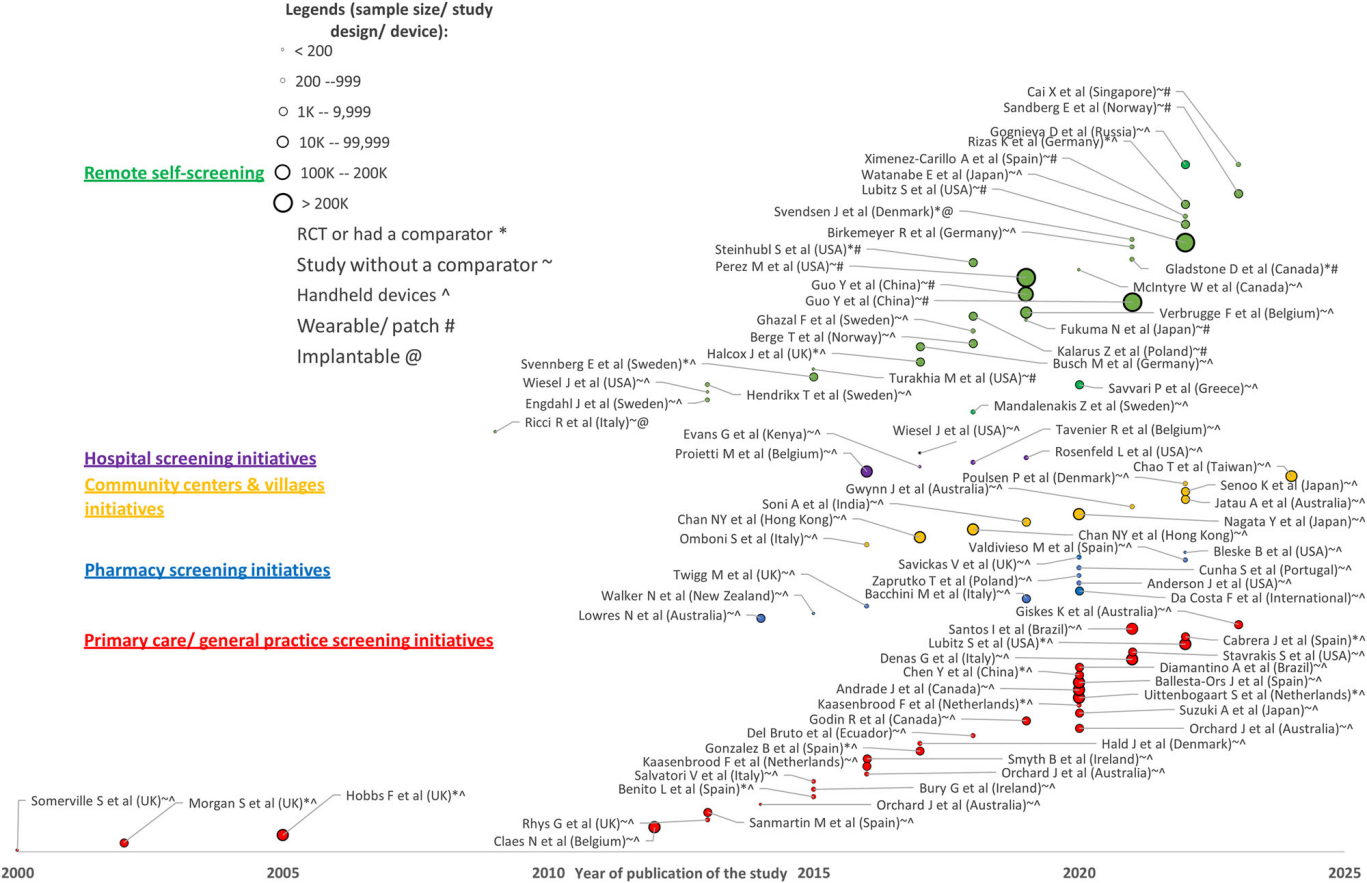
Atrial fibrillation screening studies by sample size, screening initiatives and timeline of publication. The circles’ sizes denote the study’s sample size. RCT randomised controlled trial, denoted by *. Study without a comparator is denoted by ~. Studies that applied handheld devices are denoted by ^, wearable or patch devices are denoted by #, and implantable devices are denoted by @.

**Fig. 4:**
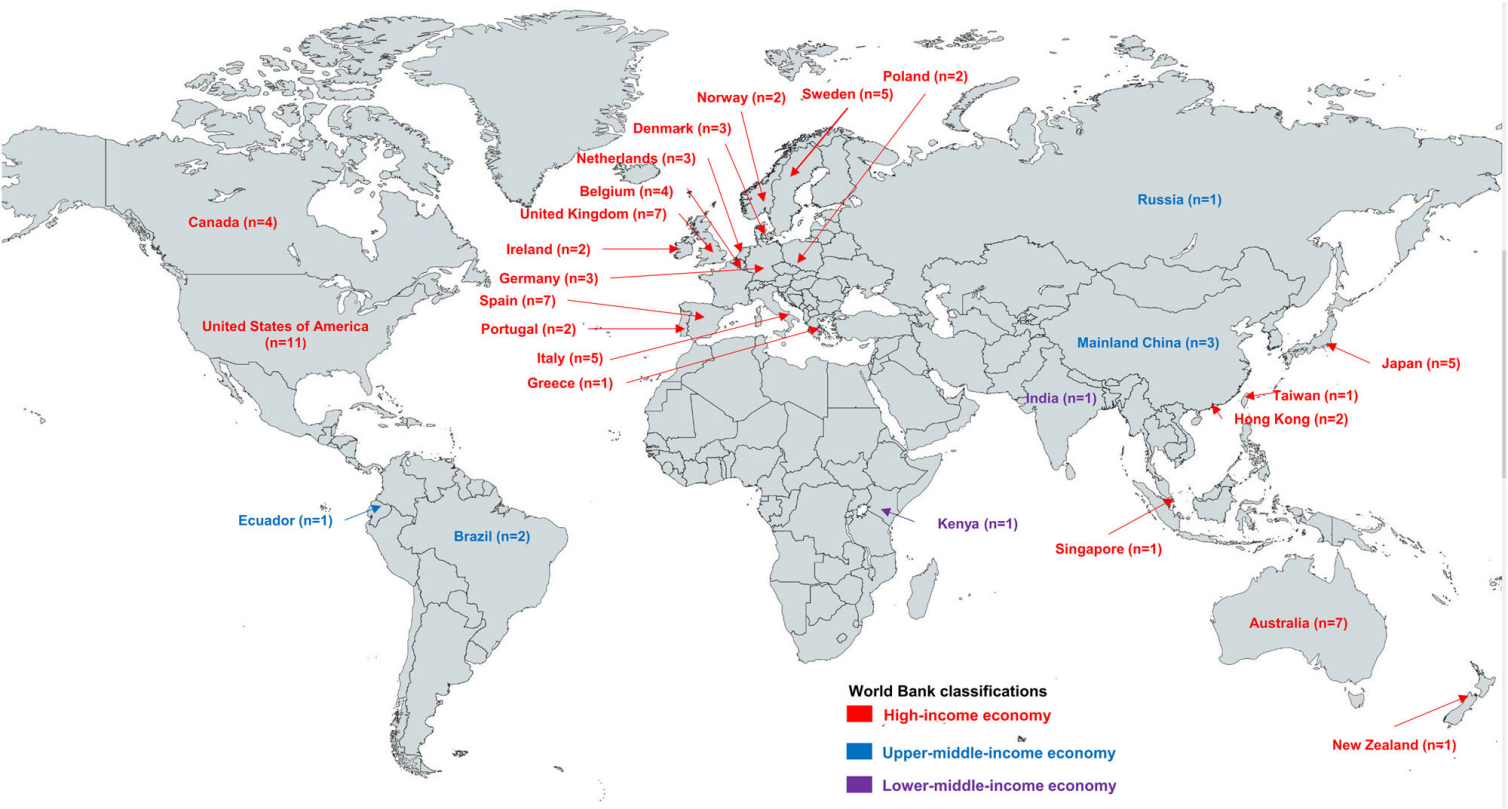
World map of atrial fibrillation screening studies. High-income countries/regions are indicated in red font, upper-middle-income countries/regions are in blue, and lower-middle-income countries/regions are in purple.

A total of 1767 abstracts were screened, 138 full-texts were reviewed, and 87 studies were identified as meeting the inclusion criteria from the last 24 years, with the majority of these studies conducted after 2010 (Fig. 3). Based on World Bank classifications^26^, 78 (90%) studies were conducted in high-income economies, 7 (8%) in upper-middle-income economies, and 2 (2%) in lower-middle-income economies (Fig. 4).

### Study design and screening initiatives

Fifteen of the studies (17%) were either randomized control trials (RCT) or had a comparator group (of these, 8 of 9 studies in primary care were RCTs, and all 6 remote monitoring studies were RCTs) (Table 1), and 72 (83%) were without a comparator group (Table 2).

Most studies examined community screening initiatives conducted in primary care/ general practice (*n* = 31)^27–61^, pharmacy (*n* = 11)^62–73^, and community centers and villages (*n* = 10)^74–84^. Several community initiatives studies were classified as ‘remote self-screening’ (*n* = 30)^85–117^, indicating that they were not connected to a community health infrastructure and relied on participants self-screening at home or elsewhere in the community and sending their ECG data to a monitoring center. The first population-based remote self-screening study (*n* = 7173) was conducted in 2015 (STROKESTOP)^112^. Some initiatives were very large, including nearly half a million individuals^102,105^ and 2.8 million individuals^95^, but only 6 of the 30 remote self-screening studies had comparator groups. A very small number of studies described screening initiatives in institutions of hospitals (*n* = 4)^118–121^ and nursing home (*n* = 1) (Fig. 3)^122^.

### Study population and sampling approach

The majority (*n* = 72/87, 83%) of studies identified their target population by a specific age group, 3 studies targeted people from more than one age group based on the presence of risk factors^70,123^ and geographical location^76^, and 12 studies did not specify age criteria. Among the 72 studies, 45 (63%) targeted older people with minimal eligible age from 65 to 80 years, while 27 (37%) targeted people with minimal eligible age from 18 to 60 years (see Supplementary Fig. 1).

Twenty-one studies used one or more risk factors, in addition to age criteria, to further target their AF screening program, with all of these studies using hypertension (21/21) as one of the risk factors, and many required patients to have one or more risk factors from a list including heart failure (18/21), diabetes (18/21), stroke or transient ischemic attack (13/21), peripheral vascular diseases (8/21), ischemic heart disease/ coronary artery disease (7/21), sleep apnea (4/21), valvular heart disease (3/21), and chronic obstructive pulmonary disease (3/21). The sampling method fitted an opportunistic approach in 56 studies (64%) and a systematic approach in 31 (36%) studies (Tables 1 and 2).

### Screening device

The screening devices used in the studies were handheld devices (*n* = 72, 83%), wearable devices (*n* = 13, 15%) and implantable devices (*n* = 2, 2%). Most primary care studies used handheld devices. Wearable and implantable ECG devices were mainly used in remote self-screening studies and mostly after the year 2015 (Fig. 3). Handheld devices included: single-lead rhythm trace devices (*n* = 43), 12-lead ECG (*n* = 13), sphygmomanometer embedded with AF-detecting algorithm (*n* = 10), 3-lead ECG (*n* = 1), photoplethysmography (PPG) app interface with smartphones (*n* = 3), and Holter (*n* = 2). Wearable devices included: ECG patch (*n* = 6); watches/ wristbands using PPG sensors (*n* = 4); ECG vest/band (*n* = 3); and implantable device/ loop recorder (*n* = 2) (Tables 1 and 2). The use of handheld and wearable screening devices by screening initiatives is shown in Table 3.

**Table 3:** Screening initiative, technology, and evaluation approach

	Community health initiatives			Other initiatives	
Number of studies (*N*)	GP *N* = 31	Pharmacy *N* = 11	Community centers *N* = 10	Hospital *N* = 5	Remote self-screening *N* = 30
Country/region *n* (%):					
High-income	27 (87)	11 (100)	9 (90)	4 (80)	27 (90)
Upper-middle	4 (13)	0 (0)	0 (0)	0 (0)	3 (10)
Lower-middle	0 (0)	0 (0)	1 (10)	1 (20)	0 (0)
RCT/Trial with a comparator *n* (%):					
Yes	9 (29)	0 (0)	0 (0)	0 (0)	6 (20)
No	22 (71)	11 (100)	10 (100)	5 (100)	24 (80)
Screening device *n* (%):					
Handheld	30 (97)	11 (100)	10 (100)	5 (100)	16 (53)
Wearable/patch	1 (3)	0 (0)	0 (0)	0 (0)	12 (40)
Implantable	0 (0)	0 (0)	0 (0)	0 (0)	2 (7)
Implementation evaluation: Yes *n* (%)	6 (19)	4 (36)	0 (0)	1 (20)	8 (27)
Method (*n*):					
Survey	2	1	0	1	8
Interview	4	3	0	0	0

*RCT* randomized control trial.

### AF detection rates

Of the 15 studies with intervention and comparator groups targeting participants ≥50 years, 9 were conducted in general practice (GP) or primary care settings, and 6 were remote self-screening studies. Six of the GP setting studies reported that the intervention screening had higher AF detection rates compared with usual care^29,31,32,39,46,124^, and three studies reported an insignificant difference in AF detection rates between intervention and control (usual care) groups^42,44,60^. Systematic screening approaches that targeted people ≥65 years through general practice yielded higher AF detection rates compared with usual care^29,31,41^, while there were insignificant differences in AF detection rates in opportunistic screening at general practice compared with usual care^42,44,60^. All 6 RCTs that were remote self-screening studies reported superior AF detection rates compared to control groups (i.e., usual care^92,99,107,111,112^ or delayed screening group^110^). The duration of remote self-screening ranged from using handheld ECG devices intermittently over 2 weeks^112^ to 12 months^99^, or wearing an ECG patch for continuous monitoring over 2 weeks^92^ to 4 weeks^110^, or having an implantable cardiac device continuous monitoring for a median follow-up of 64.5 months^111^ (Table 1).

Among the 72 studies without a comparator group, a wide range of new AF detection rates were reported: 18 studies reported new AF detection rates below 1%, 48 studies reported 1–10%, 5 studies reported above 10%, and one study reported AF incidence rate of 2.25% patient-years (95% CI 2.03–2.48) (Table 2).

**Fig. 5:**
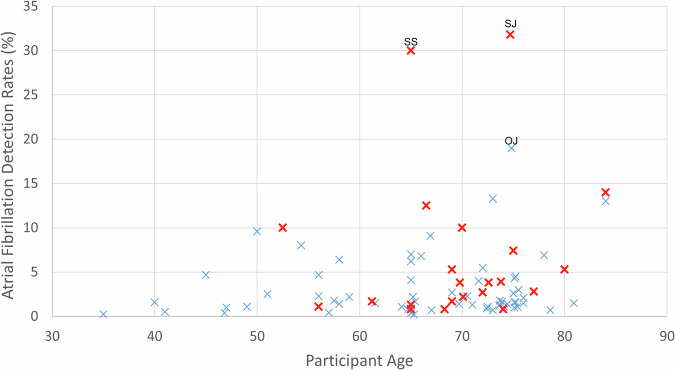
Atrial fibrillation detection rates by participant age for all screening studies. *n* = 86 (65 studies reported mean age, 11 minimal eligible age, and 10 median age). Red crosses indicate studies that included risk factors (in addition to age criteria) in the enrollment criteria, and blue crosses indicate studies with age criteria as the only enrollment criteria. SJ: Svendsen et al.^111^ screened hospital patients with at least one risk factor of stroke using continuous implanted loop recorders compared with usual care; SS: Somerville et al.^57^ included participants who had been previously diagnosed with atrial fibrillation selected from medical records at a general practice; OJ: Orchard et al.^47^ screened patients presented at a general practice and all detected atrial fibrillation patients had been previously diagnosed.

As illustrated in Fig. 5, higher AF detection rates were generally more common among older screening groups and those that targeted their screening with additional risk factors. Very high AF detection rates were reported by a few studies: Svendsen JH and colleagues reported the LOOP study with an AF detection rate of 31.8% enrolled hospital patients aged 70–90 years with at least one risk factor of stroke (*n* = 6004) and monitored them with continuous implanted loop recorders over a median duration of 64.5 months compared with usual care^111^. Somerville S and colleagues conducted a small study (*n* = 86) at a general practice with a detection rate of 30%, recruiting and screening an equal number of participants with and without a history of AF selected from the medical records; the nurses performed pulse palpation followed by 12-lead ECG if an irregular rhythm was detected^57^. One other small general practice study conducted by Orchard J and colleagues reported that receptionists and nurses screened 88 patients using handheld single-lead ECGs and found that 19% of the participants had AF, but all of them had been previously diagnosed with AF^47^.

**Fig. 6:**
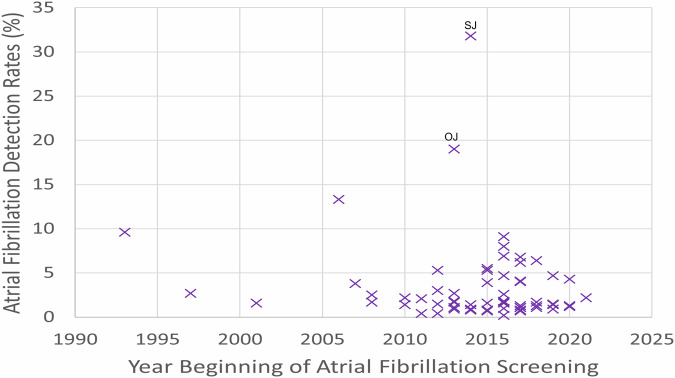
Atrial fibrillation detection rates for all screening studies that reported the year of commencement of screening. *n* = 70. SJ: Svendsen et al.^111^ screened hospital patients with at least one risk factor of stroke using continuous implanted loop recorders compared with usual care; OJ: Orchard et al.^47^ screened patients presented at a general practice and all detected atrial fibrillation patients had been previously diagnosed.

Among 70 studies that reported the date when AF screening began, 64 (91%) studies began from 2010 onwards, and the AF detection rates were mostly randomly distributed between 1% and 10% without a trend of temporal variation (Fig. 6).

### Study implementation and evaluation

Practice staff (e.g., practice nurses, GPs, and receptionists) facilitated general practice (GP)/primary care screening initiatives, with cardiologists’ support in ECG interpretation^30,39–41^. Participants’ rhythm traces were transmitted into the patient’s medical records for GPs to discuss with the patients^37,48^. There was a lack of description of the clinician’s experience reviewing rhythm traces and the process and utilization of cardiologist services in confirming and managing clinically significant rhythm abnormalities, including AF.

In pharmacy screening initiatives, participants’ rhythm traces were recorded and then remotely interpreted by cardiologists. The study team contacted participants with possible AF to see GPs for further evaluation^67,70,72^. A study involved pharmacists performing opportunistic AF screening in UK general practice during influenza vaccination^69^. A few studies involved pharmacy students performing AF screening in health promotion fairs in the community; in these studies, students handed participants a card with the results of the device’s automatic interpretation to take to their usual healthcare providers^62,64^.

Atrial fibrillation screening initiatives in the community involved setting up screening facilities in community centers^74,75,79,81^. The research team recorded participants’ rhythm traces, which were stored in a virtual portal and reviewed by the researchers 2–4 weeks later. Then, the researchers notified the participants of their AF diagnoses^74,75^. A few community screening initiatives involved sending health workers (or trained personnel) to remote rural villages to screen people using mobile technologies^82,84^. The participants’ rhythm traces were interpreted by cardiologists, and AF findings were transmitted to the participants’ GPs to consider initiation of therapy^81^.

In hospital screening initiatives, patients admitted to the hospitals were opportunistically recruited to participate in AF screening^118,120,121^. In addition, screening stations were set up in hospitals to screen eligible individuals from the community recruited through an AF awareness campaign advertised on social media. Participants with screen-diagnosed AF were referred to consult their general practitioner or cardiologist. However, the process and mode of communication were not detailed^119^. In hospital hypertension clinics and cardiologist offices, clinicians opportunistically performed triaging screening on patients, providing the screening devices to patients to perform remote screening and then re-assessing patients upon returning the screening devices. These clinician-initiated patient self-screening studies were categorized as remote self-screening because the patients performed self-screening remotely^109^. Generally, remote self-screening involved the use of various screening devices where rhythm traces were remotely reviewed by the research team^85,87,89,103,112,116^, or the research team subcontracted the ECG interpretation to paid services by another organization^99^.

Nineteen (19/87, 22%) of the studies reported an evaluation of the implementation of the screening programs, mainly (12 studies) using survey questionnaires^30,38,41,44,69,94,97,99,105,108,109,117,118^ and 7 studies conducted interviews^45,47–49,64,66–68^. Surveys were mainly applied in remote self-screening studies evaluating participant acceptability, usability and satisfaction with the screening, while interviews were mainly conducted in general practice and pharmacy screening initiatives (Table 3). Surveys in Apple Heart Study^105^ and Fitbit Heart Study^102,125^ found that 57% of participants in Apple Heart Study^105^ and 24% of respondents in Fitbit Heart Study^102^ visited their usual healthcare providers outside the research study when they received irregular rhythm notifications. Generally, surveys of remote self-screening reported good acceptability and usability of the screening^94,99,108^. For example, researchers surveyed participants and obtained a System Usability Score^126^ of 85 (a score above 68 is generally considered acceptable), indicating high usability of the ECG patch worn by participants aged 65 years and older^108^.

In the included studies, researchers applied thematic analysis to semi-structured interviews and identified barriers, e.g. perception that pharmacist’s roles do not include screening, lack of time to engage with customers and difficulty in integrating screening in the workflow, and enablers, e.g. incorporating AF screening in pharmacy health promotion events and training pharmacists to use the screening devices^67^ and appointing a local “champion”, i.e. someone to facilitate the implementation of AF screening in pharmacy^66^. In addition, in assessing how to make AF screening at pharmacies sustainable, researchers identified that pharmacy students could provide sustainability of AF screening in pharmacies^64^. In general practice/primary care studies, researchers applied the Realist evaluation approach^23^ in analyzing qualitative semi-structured interviews^127^. The Realist approach has provided a more robust framework for assessing participants’ perceptions of and experience with the screening processes and generating themes from the qualitative interviews^23^. The main themes in the GP setting included a lack of resources and referral pathways in busy clinical settings as screening barriers, while enablers included the appointment of a designated person to lead and facilitate AF implementation at the point-of-care setting^45,47–49,68^.

## Discussion

This review found substantial literature on AF screening, mainly from higher-income countries/ regions, that mostly described opportunistic approaches to screening (64% of studies) targeting older patients aged 65 years and older in line with many current Guidelines^11–14^. However, over this 24-year period, less than 20% of studies had a comparator group reporting a measure of the effectiveness of the screening approach, only 14 studies were RCTs (Table 1) and only about 20% reported on some implementation measures (Table 3) with very few providing data to inform how to scale up AF screening. The studies described initiatives across various settings (primary care, pharmacy, community centers, hospital and remote screening). Handheld ECG devices were commonly used, and the screening initiative was most commonly in a location associated with existing healthcare with access to direct health professional supervision, with some describing remote self-screening (Table 3).

### Screening approaches, enablers, and barriers in primary care

Many studies (*n* = 31) were general practice/primary care screening initiatives. Primary care services are common in many countries. Among 38 countries in the Organization for Economic Co-operation and Development (OECD), on average, approximately 80% of individuals aged 15 or older reported visiting a doctor (using primary care service) at least once a year, ranging from around 65% in Sweden and the United States to 89% in France^128^. In Australia, a recent national health survey found that 90% of Australians visited their general practitioners (GPs) at least once a year^129^. This healthcare utilization was even higher among older people. In 2019–2020, 95% of people aged ≥65 years saw a GP at least once yearly^130^. GP visits provide occasions for opportunistic AF screening, such as palpating the patient’s pulse and acquiring an ECG if clinically indicated^41,124^. However, the frequency of opportunistic AF screening occurring in general practice is uncertain. A survey of GPs in Australia found that raising GPs’ awareness of AF screening and improving their confidence in ECG interpretation may increase AF screening^131^. In the SAFE Study conducted in the UK^41^, GPs and practice nurses in the intervention group received education about the importance of AF screening and ECG interpretation, and they palpated patients’ pulses, followed by acquiring 12-lead ECGs if irregular pulses were detected. However, the SAFE Study reported only a modest increase in AF detection rate (difference of 0.59%, 95% CI 0.20–0.98%) between the intervention and control (usual standard care) groups.

Most studies in this review utilized mobile devices. Some research indicates that using single-lead handheld ECG devices to detect AF is more accurate than pulse palpation. A recent study involving 6159 participants reported that the sensitivity of pulse palpation was 78.6% and positive predictive value (PPV) was only 4.8%^132^, while handheld single-lead ECG devices generally have high accuracy (sensitivity of 90% and specificity of 99%) in detecting AF^133^. However, existing data also indicate that the uptake of AF screening among GPs is low, partly due to time pressure and lack of resources^47,127^. Some studies have described potential enablers such as identifying practice staff resources and including AF screening in their roles^43,47,49^, and designating “AF-screening champions”^49^ in practice. When practice administrative staff were encouraged to be involved, some studies suggested their reluctance because tasks were beyond their traditional scope of duties^47^. For practice nurses, AF screening was more easily integrated into their workflow^49^. While several studies have focused on GPs (primary care providers) implementing AF screening, this may not be practicable in some countries. Primary care is increasingly under strain. Nonetheless, it may be feasible for primary care providers to focus screening of AF in the highest risk groups, such as older patients, as part of an overall health check. Notably, financial viability was reported as important to implementation, with some indicating the need for financial incentives to make screening feasible^42,48,134^.

This review provided additional insights into other novel approaches to implementing AF screening within health practice. For example, optimizing the use of patients’ waiting time was examined in one study^37^ involving the setting up self-screening stations in the waiting room. Patients placed their fingers on the designated contact points of single-lead Kardia ECG devices; the automated ECG results were transmitted to the patient’s medical records, and the GP could discuss the results with them. This study reported the general acceptability of this screening approach. However, some older patients reported needing assistance to use the devices and touching shared screening devices could be a barrier (particularly in the context of the COVID-19 pandemic)^37^. This approach might be feasible but requires a sustainable process for GPs to interpret unclassified ECGs (i.e., ECGs that could not be interpreted by the device’s automated algorithms), suspected AF and other abnormalities, and to have timely access to referral pathways when an abnormality was detected. A qualitative evaluation with GPs conducted in the UK recently highlighted that the lack of an established protocol or a referral pathway to manage ECG abnormalities was a barrier to implementing AF screening^135^. A survey of 588 healthcare providers (77% from Europe, 14% from Asia/ Oceania and 8% from North or South America) conducted by the AF-SCREEN International collaboration reported that there is a need to better define an appropriate mechanism for managing screen-detected AF^136^.

Pharmacies are commonly visited venues by the public. Providing handheld single-lead ECG devices to pharmacists, training them to perform AF screening and tasking them to refer suspected abnormalities to GPs, were reported to be feasible in a study involving 1000 pharmacy consumers^68^. This approach optimises the interaction between pharmacies and GPs, which is essential in many aspects, such as medication reviews and detecting high blood pressure at pharmacies. Nonetheless, for large-scale implementation, the issues of resources (training large numbers of pharmacists and providing them with screening devices) and providing financial incentives for pharmacies to sustain AF screening would all need to be addressed. However, it was encouraging to read that AF screening was included in the pharmacy curriculum of a university, and their pharmacy students were trained to perform AF screening using handheld single-lead ECG devices as a pharmacy community service^62,64^.

### Remote self-screening

A significant number of studies (30/87) focused on self-screening and remote monitoring approaches to AF screening; most of these were in high-income countries, with roughly half using a mobile handheld device and the other half using a wearable device (Table 3) and 6 studies compared the self-screening approach to usual care, and all showed superior AF diagnosis rates compared to usual care (Table 1). For example, in Sweden, the STROKESTOP study involved 7173 people aged 75–76 years in self-recording ECGs using handheld ECG devices remotely twice daily over 2 weeks, and the study reported a net benefit of screening and treatment compared to usual care^112^. This population-based screening systematically identified eligible participants and invited them to participate, which was different from opportunistic point-of-care screening. This type of systematic screening offers equitable access to eligible populations, while opportunistic point-of-care screening is subjective to the context of each health visit and the interaction between the clinician and patient.

Remote monitoring typically involves participant-led self-screening, and a central team (including trained personnel/ technicians/ research nurses and cardiologists) that remotely monitors, reviews and interprets the participants’ ECGs^87,106,111,112,137^. One study subcontracted ECG interpretation to an external service provider^99^, and other studies incorporated a two-stage diagnostic approach. When an irregular pulse was detected by the smart devices’ photoplethysmography sensors, participants were sent an ECG patch to be worn for a week, which was analyzed upon return^102,105^. This type of market-driven wearables and screening of broader, often low-risk populations, potentially stresses the already busy general practitioners who must address potential abnormalities, which are often false positives. Additionally, it poses challenges to providing integrated continual care, including how to fund remote ECG interpretation services, ensure patients with abnormal findings access the necessary care, and integrate the screening results with patient medical records in their usual medical practice.

Remote self-screening as a triage to further investigations was an approach applied in the “mobile Atrial Fibrillation App” (mAFA) I and mAFA II trials”^95–98,138^, Apple Heart Study^105^, and Fitbit Heart Study^102,125^. In the mAFA I & II trials, participants with “possible AF” detected by the Huawei watches or wristbands were further investigated using ECG or Holter monitors through the designated mAFA telecare team. The integrated management included anticoagulants for stroke prevention, better symptom management, cardiovascular and comorbidity risk management, and educational programs. However, modest rates of effective follow-up were reported (Table 1)^94,98,138,139^. In Apple Heart Study^105^ and Fitbit Heart Study^102,125^, when the wearable devices detected irregular heart rhythms, the participants were provided with a telemedicine visit and mailed an ECG patch to be worn for a week. The researchers reported many participants visited their usual healthcare providers outside the research study when they received irregular rhythm notifications, implying that this could be patients’ usual health-seeking behavior when a potential abnormality with their health was detected. Therefore, the remote monitoring systems should be integrated into the patient’s usual primary care providers to ensure continuity of care. Despite the surveys showing the general acceptability and usability of this screening approach^94,99,108^, there was a lack of in-depth qualitative process evaluation using a robust framework^22–25^ to evaluate this model of screening.

### Reaching out to the communities

In 10 (12%) studies, community screening occurred in various settings and involved a dedicated in-person screening team (e.g., screening booths in communities or screening campaigns in villages). In Hong Kong, screening stations in community centers were set up to screen eligible people in the community and the rhythm traces were interpreted by the researchers remotely^74,75^. This screening model could be feasible in some settings for people who can access screening venues, but it may miss out on people in more remote areas. Some studies did describe approaches to integrating AF screening in rural health facilities to make screening more accessible to rural residents, including Indigenous communities^58,77^. In a study in India, health workers visited and screened villagers using handheld single-lead ECG devices^84^, and in Denmark, caregivers visited older people at homes and screened them using mobile devices^82^.

### Evaluation of screening implementation

Only a few studies included some components of a structured implementation evaluation. This was most commonly in the form of implementation evaluation surveys among intervention screening program participants, addressing the acceptability and usability of the screening programs^94,99,108^. However, most survey evaluations did not report the contextual enablers and barriers to implementation because they did not have an evaluation framework^22,25,140^. Only a small percentage (7/87, 8%) of studies included in-depth interviews of participants and fewer in-depth interviews of personnel involved in the screening program implementation. Such qualitative evaluations could have elicited further information on enablers and barriers to implementing AF screening programs^45,47–49,68^. The Realist^23^ or Critical Realism^24^ evaluation approach, where the implementation process, context, mechanism of impact and outcomes were examined, could be adopted as in some selected studies^48,127,141,142^. Generally, there is a lack of application of evaluation frameworks, such as the Medical Research Council Guidelines on Process Evaluation^22^ and the RE-AIM^25^, and a lack of transparency of refinements to interview processes^141^.

This review did not aim to examine screening costs, which entails a different literature search strategy. Cost-effectiveness is important in considering the future implementation of AF screening programs. This review did identify a spectrum of screening devices ranging from handheld devices to wearable patches being used, and the unit costs of the various devices do vary^143^, and the cost implications are contextual to the country and application settings^144^. In a study using nurses to perform pulse palpation followed by a 12-lead ECG when an irregular rhythm was detected, Morgan S et al. estimated £6 per consultation with a practice nurse, resulting in a cost per AF case detected of £186 (95% CI = £138–£300) for a yield of 4.5% AF detection rate using systematic screening compared with 1.3% employing opportunistic screening^46^. Using a similar pulse palpation approach, Hobbs et al. estimated £337 for each additional AF case detected by opportunistic screening compared to usual care, for a yield of 1.63% (opportunistic screening) versus 1.04% (usual care) AF detection rates^41^. Using handheld single-lead ECG devices to screen participants ≥65 years old twice weekly over a year yielded an almost fourfold increase in AF detection compared with usual care, and the estimated cost was £8255 per additional AF diagnosis^99^. Studies that applied devices that could continuously screen for months, such as the study using implantable loop recorders over a median follow-up of 64.5 months, would entail providing devices and interrogating large amounts of data and, as such, would be far more costly^111^. Continuous remote monitoring could yield a higher AF detection rate at a higher cost but may identify more low-risk AF patients. Moreover, there is a lack of reports on the cost-effectiveness of such studies.

### Strengths and limitations

A structured literature search strategy (Supplementary Table 1) was used, yielding a spectrum of AF screening across various settings (general practice, pharmacy, community centers, villages, hospitals, and remote screening) over a 24-year period. This provided insights into the utilities of various technologies and screening approaches. However, the search was limited to one database (PubMed). Systematic review approaches of replication of search and data extraction using multiple databases were not applied.

## Conclusions

The current review has identified and synthesized the findings of 87 studies on AF screening approaches, technology, and program implementation. The findings demonstrate a large breadth of approaches to implementing AF screening but, somewhat surprisingly, lack strong data on the effectiveness of approaches with few RCTs or studies with comparator groups and very limited information to inform implementation or scale-up beyond the research setting. Despite the recommendations in many guidelines, the relative dearth of information on how to implement AF screening may be a reason for the lack of widespread implementation of AF screening. This review suggests that primary care is a feasible location for implementing AF screening, at least in some existing healthcare systems, with additional resources including relatively low-cost mobile devices and designated personnel to lead AF screening that targets individuals 65 years and older. Yet further research to examine other community approaches, especially for settings in which primary care services are not integrated or accessible, is needed. More recent studies show that a potential alternative to primary care screening is the implementation of remote screening programs, but remote self-screening programs need careful evaluation amongst older populations. However, there is limited remote screening research with comparator groups that target older people. Similarly, there is limited evaluation of the potential adverse impact of unguided population-wide screening on an already over-burdened healthcare system (and especially the primary care sector). The lack of integration of these remote screening initiatives into existing healthcare systems could hinder their implementation. Finally, this review provides limited data on the financial models and cost-effectiveness of AF screening initiatives, and further research is needed to establish cost-effective approaches to scale up AF screening. AF screening is recommended by many guidelines, and this review synthesises what we know from the last 24 years but also highlights the practical information gaps that will help implement AF screening and integration into existing health systems in the future.

## Supplementary Information

Supplementary materials
