# Author Correction: Global implementation and evaluation of atrial fibrillation screening in the past two decades – a narrative review

**DOI:** 10.1038/s44325-025-00066-6

**Published:** 2025-06-27

**Authors:** Kam Cheong Wong, Tu N. Nguyen, Clara K. Chow

**Affiliations:** 1 Westmead Applied Research Centre, Sydney Medical School, Faculty of Medicine and Health, The University of Sydney, Sydney, NSW Australia; 2 Westmead Clinical School, Faculty of Medicine and Health, The University of Sydney, Westmead, NSW Australia; 3 Bathurst Rural Clinical School, School of Medicine, Western Sydney University, Bathurst, NSW Australia; 4 School of Rural Health, Faculty of Medicine and Health, The University of Sydney, Orange, NSW Australia; 5 The George Institute for Global Health, Sydney, NSW Australia; 6 Department of Cardiology, Westmead Hospital, Westmead, NSW Australia; 7 Charles Perkins Centre, The University of Sydney, Sydney, NSW Australia

**Keywords:** Cardiology, Health care

Correction to: *npj Cardiovascular Health* 10.1038/s44325-024-00014-w, published online 2 September 2024

In this article the Competing Interests statement was incorrect and should have been ‘Author Clara K. Chow is Associate Editor at *npj Cardiovascular Health*. Clara K. Chow was not involved in the journal’s review of, or decisions related to, this manuscript. Author Tu N. Nguyen is an Editorial Board Member at the journal.’ The original article has been corrected.

