# Supplementary material for: Global implementation and evaluation of atrial fibrillation screening in the past two decades – a narrative review
Source: NPJ Cardiovasc Health. 2024 Sep 2;1:17. doi: 10.1038/s44325-024-00014-w (PMC12912355; doi:10.1038/s44325-024-00014-w)

Supplementary Materials

Supplementary Table 1 Literature Search Strategies

Searched PubMed (National Library of Medicine): ((atrial fibrillation[Title]) OR (auricular fibrillation[Title]) OR (atrial flutter[Title])) AND ((screen[Title]) OR (screening[Title]) OR (search[Title]) OR (searching[Title]) OR (detect[Title]) OR (detecting[Title]) OR (detection[Title]) OR (diagnose[Title]) OR (diagnosing[Title]) OR (diagnosis[Title]) OR (diagnoses[Title]) OR (Identify[Title]) OR (Identifying[Title]) OR (Identification[Title])) - Saved search Filters: Humans, English, from 2000/1/1 - 2024/1/18.

Search	Query	Results
#1	atrial fibrillation[Title]	54,387
#2	auricular fibrillation[Title]	808
#3	atrial flutter[Title]	2,766
#4	screen[Title]	19,308
#5	screening[Title]	195,537
#6	search[Title]	32,375
#7	searching[Title]	6,944
#8	detect[Title]	21,818
#9	detecting[Title]	31,309
#10	detection[Title]	350,169
#11	diagnose[Title]	6,374
#12	diagnosing[Title]	15,844
#13	diagnosis[Title]	439,402
#14	diagnoses[Title]	9,560
#15	identify[Title]	31,452
#16	identifying[Title]	37,314
#17	identification[Title]	259,031
#18	#1 OR #2 OR #3	57,429
#19	#4 OR #5 OR #6 OR #7 OR #8 OR #9 OR #10 OR #11 OR #12 OR #13 OR #14 OR #15 OR #16 OR #17	1,419,693
#20	#18 AND #19	2,447
#21	#18 AND #19 Filters: Humans	1,937
#22	#18 AND #19 Filters: Humans, English	1,837
#23	Search: #18 AND #19 Filters: Humans, English, from 2000/1/1 - 2024/1/18	1,767

Supplementary Figure 1 Number of atrial fibrillation screening studies by minimal eligible age criteria

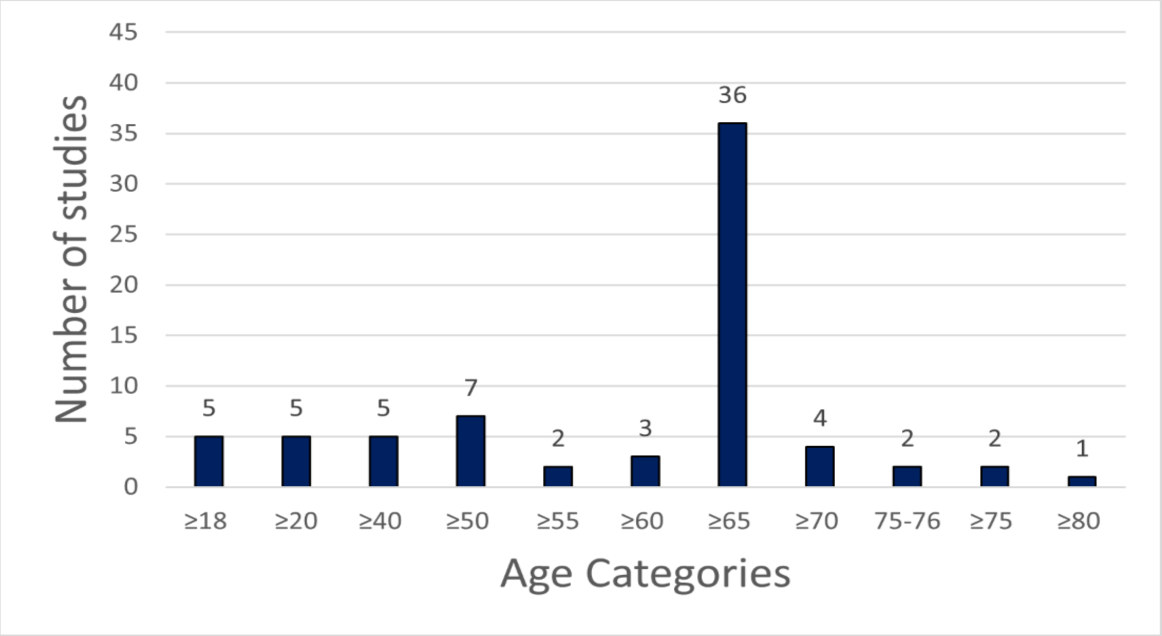

Supplement: Supplementary file 1 — Supplementary materials [file 44325_2024_14_MOESM1_ESM.pdf]
